# Specific *Dioscorea* Phytoextracts Enhance Potency of TCL-Loaded DC-Based Cancer Vaccines

**DOI:** 10.1155/2013/932040

**Published:** 2013-07-07

**Authors:** Wei-Ting Chang, Hui-Ming Chen, Shu-Yi Yin, Yung-Hsiang Chen, Chih-Chun Wen, Wen-Chi Wei, Phoency Lai, Cheng-Hsin Wang, Ning-Sun Yang

**Affiliations:** ^1^Department of Food and Nutrition, Providence University, No. 200, Section 7, Taiwan Boulevard, Shalu District, Taichung 43301, Taiwan; ^2^Institute of Agricultural Biotechnology Research Center, Academia Sinica, No. 128, Section 2, Academia Road, Nankang, Taipei 11529, Taiwan

## Abstract

*Dioscorea* tuber phytoextracts can confer immunomodulatory activities *ex vivo* and improve regeneration of bone marrow cells *in vivo*. In present study, we evaluated specific *Dioscorea* phytoextracts for use *ex vivo* as a bone-marrow-derived dendritic cell- (DC-) based vaccine adjuvant for cancer immunotherapy. Fractionated *Dioscorea* extracts (DsII) were assayed for their effect on maturation and functions of DC *ex vivo* and antimelanoma activity of DC-based vaccine *in vivo*. The phytoextract from 50–75% ethanol-precipitated fraction of *Dioscorea alata* var. *purpurea* Tainung no. 5 tuber, designated as DsII-TN5, showed a strong augmentation of tumor cell lysate- (TCL-) loaded DC-mediated activation of T-cell proliferation. DsII-TN5 stimulated the expression of CD40, CD80, CD86, and IL-1**β** in TCL-loaded DCs and downregulated the expression of TGF-**β**1. DC vaccines prepared by a specific schema (TCL (2 h) + LPS (22 h)) showed the strongest antitumor activity. DsII-TN5 as a DC vaccine adjuvant showed strong antimelanoma activity and reduced myeloid-derived suppressor cell (MDSC) population in tested mice. DsII-TN5 can also activate DCs to enhance Th1- and Th17-related cytokine expressions. Biochemical analysis showed that DsII-TN5 consists mainly of polysaccharides containing a high level (53%) of mannose residues. We suggest that DsII-TN5 may have potential for future application as a potent, cost-effective adjuvant for DC-based cancer vaccines.

## 1. Introduction

Therapeutic vaccines based on dendritic cell- (DC-) carrying tumor-associated antigens (TAAs) have emerged as a promising, yet challenging strategy to elicit and/or augment an immune response against specific malignancies [1]. Tumor cell lysate- (TCL-) loaded DC vaccines have demonstrated strong induction of cytotoxic activity of splenocytes against target tumor cells, effective retardation of tumor growth, and significant increase in survival of tested mice [2]. Recently, the US Food and Drug Administration approved a DC-based therapeutic vaccine for the treatment of specific prostate cancers [3].

In DC-based cancer vaccines, effective boosting of the immunogenicity of target tumor cells for recognition by DCs is considered critical for subverting the “immune escape” activities evoked by tumor cells. We have shown that combined TCL and LPS treatment can activate DCs to maturation status and enhance the priming of Th1/Th17 effector cells [2]. Recent reports, however, also indicated that TAAs can inhibit the maturation of DCs [4, 5]. Maturation of DCs is essential for effective induction of antitumor immunity. With efficacious and optimized stimulation, DCs can prime a strong tumor antigen-specific effector T-cell response. DCs are known to detect and react to conserved pathogen-associated molecular patterns (PAMPs) through pattern-recognition receptors (PRRs) [6], including Toll-like receptors (TLRs) [7]. Maturation of DCs can be achieved by exposure to various stimuli including cytokines and specific TLR ligands [8, 9]. However, use of cytokines as maturation reagents for DCs, for example, via use of recombinant cytokines would be very expensive effectively, precluding their use as a general public health measure, especially in nondeveloped countries. The maturation of DCs can be readily achieved though activation by LPS treatment; however, this method may induce dangerous, perhaps lethal side effects [10, 11]. Currently, due to various cytotoxicity and systemic concerns, clinical use of LPS as an adjuvant for immunotherapy is prohibited [12]. Because of the shortcomings of these known DC maturation reagents, it is important to continue to search for sources of potential efficacious adjuvants to serve as potent, safe, and cost-effective stimuli to induce full maturation of TCL-loaded DCs and allow optimal delivery of DC-based cancer vaccines. Recently, monophosphoryl lipid A (MPL) was approved by the FDA as an adjuvant. MPL, which acts as a TLR4 agonist, promotes the broadening of an immune response [13]. Another study showed that a synthetic peptide, an LPS peptide mimic, functions as a potent TLR4 agonist adjuvant [14]. Therefore, identification of new adjuvants, such as TLR agonists, that can induce inflammation like LPS, but not on a systemic scale, are of prime significance in vaccine applications. Here, we tested the possible application of immunomodulatory phytocompounds that can be readily extracted in large quantities from a traditional medicinal plant as adjuvants for cell-based cancer vaccines.

Yams (various *Dioscorea *species) are members of the monocotyledonous plant family Dioscoreaceae [15]. *Dioscorea* tuber slices are widely used in Asia as a functional food or traditional Chinese medicine [16]. Many studies including ours have shown that *Dioscorea* tubers confer a broad spectrum of biological activities, including reduction of lung metastasis in a murine melanoma model [17], analgesic and anti-inflammatory properties in mice and rats [18], and antiosteoporotic activity through driving mesenchymal stem cell differentiation for bone formation [19]. The immunological activities of specific polysaccharides and/or glycoproteins in *Dioscorea* tubers are attracting increasing attention in part due to reports of their TLR4-signaling pathway-mediated immunomodulatory and cytokine-regulation activities [20, 21]. These findings suggest that *Dioscorea* extracts may be a valuable resource for the discovery of effective but nontoxic TLR4 agonists that activate TLR4 signaling to induce immune responses [20, 21]. Our previous study also showed that *Dioscorea* phytoextracts can enhance the proliferation of murine splenocytes *ex vivo* and promote regeneration of specific progenitor cell populations in damaged bone marrow tissues of 5-fluorouracil-treated mice [22]. Mouse myeloid precursor cells and mouse bone-marrow-derived dendritic cells (BMDCs) have been used in several studies as models for research into the development of DC-based therapeutics [2, 23]. 

Here, we investigated the bioactivities of specific *Dioscorea* tuber phytocompound fractions on tested DCs and their application as adjuvants in DC-based cancer vaccines. A specific, ethanol-precipitated fraction of *Dioscorea alata* var. *purpurea* Tainung no. 5 extract, designated as DsII-TN5, effectively stimulated DC-mediated activation of T-cell proliferation and suppression of melanoma tumor growth. 

## 2. Materials and Methods

### 2.1. *Dioscorea* Phytoextract Preparation

Six *Dioscorea* plants from four species, *Dioscorea batatas *(*bat*),* D. japonica* (*jap*), *D. persimilis *(*per*), and *D. alata* were used. From the *D. alata* species, three strains, *D. alata *Tainung no. 1 (TN1), *D. alata *var.* purpurea *Tainung no. 5 (TN5), and *D. alata *var.* purpurea* zih-yu-xie-shu (zih) were used. *Dioscorea* tuber phytoextracts were prepared as previously reported [22]. Briefly, dried powders of tubers were extracted with sterilized water; then the supernatants of specific extract preparations were pooled as the crude extract fraction. This fraction was further extracted stepwise with 50%, 75%, and 87.5% (V/V) ethanol. The resultant 50–75% ethanol-partitioned fractions, designated as DsII, were lyophilized and stored at room temperature until use. 

### 2.2. Cell Lines

Mouse B16F10 (B16) melanoma cell line was purchased from American Type Culture Collection (ATCC; Manassas, VA, USA). Tumor cell cultures were maintained in Dulbecco's modified Eagle's medium supplemented with 10% fetal bovine serum (FBS), 1.5 g/L sodium bicarbonate, 100 *μ*g/mL streptomycin, 100 unit/mL penicillin, and 2 mM L-glutamine. 

### 2.3. Mice

Female C57BL/6JNarl mice (6–8 weeks old) were purchased from the National Laboratory Animal Breeding and Research Center, Taipei, Taiwan. All mice were maintained in a laminar airflow cabinet kept at 24 ± 2°C and 40–70% humidity with 12 h light/dark cycles under specific pathogen-free conditions. All facilities and animal experimental protocols were approved by Academia Sinica Institutional Animal Care and Utilization Committee.

### 2.4. Mouse Bone-Marrow-Derived Dendritic Cells (BMDCs)

Mouse bone-marrow-derived DCs were generated and modified as previously described [23]. Briefly, bone marrow tissues were collected from the femurs and tibiae of C57BL/6 mice. Erythrocytes were removed from the bone marrow cells by ACK lysis buffer and plated in RPMI 1640 culture medium supplemented with 20 ng/mL GM-CSF, 10% FBS, 100 *μ*M nonessential amino acids, 50 *μ*M 2-mercaptoethanol, 100 *μ*g/mL streptomycin, and 100 unit/mL penicillin in a humidified 5% CO_2_ incubator at 37°C. On day 2, two-thirds of the original medium were replaced by 30 mL fresh medium supplemented with 20 ng/mL GM-CSF. On day 5, the floating cells were gently removed and the fresh medium was replenished with 20 ng/mL GM-CSF and 20 ng/mL IL-4. On day 7, nonadherent and loosely adherent DCs in culture were harvested. DCs generated in this manner were found mainly as immature DCs and they displayed typical morphologic features of DCs. The purity of these DC populations was determined by flow cytometry analysis. The procedure routinely resulted in ≥85% CD11c^+^ DCs.

### 2.5. Preparation of Tumor Cell Lysates (TCLs)

Tumor cell lysates of B16 melanoma were prepared as previously described [24]. In brief, cells were resuspended in PBS and subjected to four freeze-thaw cycles. Cell lysates were then centrifuged for 20 min at 13000 rpm, and the supernatant was used as the source of tumor antigen. TCL protein concentrations were determined using the BCA assay (Pierce, Rockford, IL, USA).

### 2.6. Stimulation of TCL-Pulsed DCs by Treatment with DsII Extracts

BMDCs (3 × 10^6^) were loaded with TCL containing 50 *μ*g protein/mL and then incubated for 2 h. *Dioscorea* tuber phytoextract or LPS was added to the culture medium and used as stimulators, and DCs were incubated for another 22 h. The stimuli were made by serial dilutions at concentrations of 10, 50, 100, 200, 500, and 1000 *μ*g/mL of DsII-TN5 or 1 *μ*g/mL of LPS, and the expression of cell-surface molecules (i.e., CD40, CD80, CD86, and CD11c) (BD Pharmingen, San Diego, CA, USA) and stimulation of T-cell proliferation were determined by flow cytometer and BrdU Incorporation, respectively. The corresponding culture media were collected to determine the expression of cytokines (i.e., IL-1*β*, TGF-*β*1, IL12p70, and IL-10) (R&D Systems, Minneapolis, MN, USA) by ELISA, according to the manufacturer's instructions.

### 2.7. T-Cell Proliferation Assays

Splenic T cells to be used as responders were isolated by magnetic activated cell sorting (MACS) selection of splenocytes with CD4 and CD8 microbeads (Miltenyi Biotech; >98% purity and >98% viability). Cocultivation of the DC and T-cell was performed as previously described [25]. T cells (10^5^) were cocultured with BMDCs at the DC/T ratios 1 : 1, 1 : 10, 1 : 100, and 1 : 1000 for 4 days, and then BMDC-induced T-cell proliferation was determined.

### 2.8. DC-Mediated T-Cell Polarization Assay

A total of 2 × 10^5^ CD4^+^ T cells were cultured with 5 × 10^4^ DsII-TN5-treated DCs (1 : 4 DC/T cells ratio) in RPMI media with 10% FBS for 4 days. Cells were stimulated with 50 ng/mL phorbol 12-myristate 13-acetate (PMA) and 1000 ng/mL ionomycin for 6 h. DC-CD4 T cells from the cocultivation media were harvested and assayed for production of Th1-, Th2-, and Th-17-related cytokines (i.e., IFN-*γ*, IL-4, and IL-17A) by ELISA (eBioscience and R&D).

### 2.9. Tumor Inoculation, Vaccination, and Tumor Challenge

For tumor inoculation, B16 tumor cells (1 × 10^5^/mouse) were injected subcutaneously into the right flank of mice. When tumor volume reached 80 mm^3^, tumor-bearing mice were vaccinated with different preparations of TCL-loaded DCs. For optimization of therapeutic immunization, test mice were divided into four experimental groups (five mice/group): (1) TCL (12 h) + LPS (12 h), (2) TCL (2 h) + LPS (12 h), (3) TCL (2 h) + LPS (22 h), and (4) LPS (2 h) + TCL (22 h). See Figure 5(a) for schema. The times indicated are the time period of incubation of treatment with tested DCs. To investigate the adjuvant effect of DsII-TN5 on TCL-loaded DCs in the B16 melanoma model, mice were divided into seven experimental groups (five mice/group): (1) PBS (untreated control), (2) iDC, (3) iDC + TCL, (4) iDC + TCL + DsII-TN5 (50 *μ*g/mL), (5) iDC + TCL + DsII-TN5 (100 *μ*g/mL), (6) iDC + TCL + DsII-TN5 (200 *μ*g/mL), and (7) iDC + TCL + LPS (positive control). These mouse groups were vaccinated with both priming and booster vaccinations. See Figure 5(b) for schema. Ten days after the second booster, peripheral blood samples were collected from immunized mice. After elimination of erythrocytes from test samples, the total population of white (leukocyte) cells were assayed for the population of myeloid-derived suppressor cells (MDSCs; CD11b^+^Gr-1^+^ cells), using flow cytometry. Tumors were measured and calculated by the formula: volume = length × (width)^2^/2. Survival time of tested mice was observed. 

### 2.10. Monosaccharide Composition Analysis

Composition of monosaccharides in DsII-TN5 phytoextracts was examined by high-performance anion-exchange chromatography (HPAEC) as described previously with slight modification [26]. Chromatography of DsII-TN5 was performed in a Dionex LC20 system with an electrochemical detector (model ED50) (Dionex, Sunnyvale, CA, USA). A CarboPac PA100 column in combination with a guard column (Dionex, Sunnyvale, CA, USA) was used. Eluent was obtained as a 4 : 96 (V/V) mixture of 200 mM NaOH and deionized water at 0.75 mL/minute. 

### 2.11. Statistical Analysis

Data were analyzed by one-way or two-way ANOVA. Statistical analyses were conducted with GraphPad Prism 5.0 (San Diego, CA, USA). Differences in mouse survival time and rate were determined by a log-rank (Mantel-Cox) test of the Kaplan-Meier survival curves. All statistical tests were two sided. A *P* value of less than .05 was considered significant (*P < .05; **P < .01; ***P < .001; ns: no significance).

## 3. Results

### 3.1. DsII-TN5 Is Most Effective in Stimulating TCL-Loaded DCs to Activate Proliferation of Syngeneic T Cells

**Figure 1:**
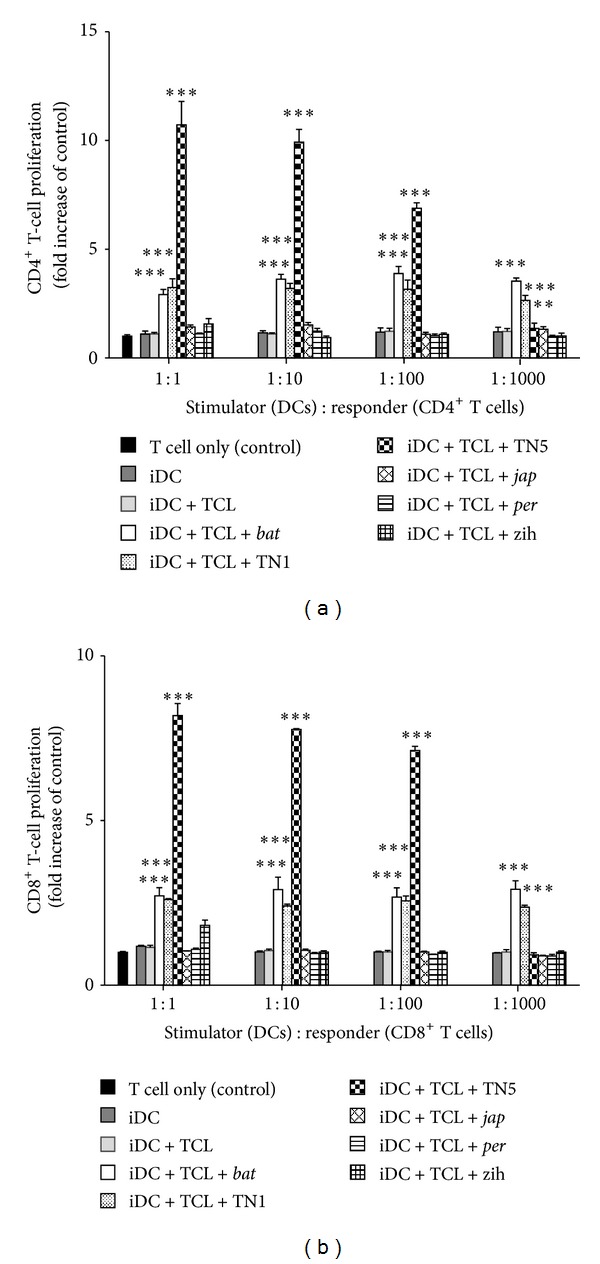
Effect of DsII from different *Dioscorea* species or strains on DC-activated T-cell proliferation. TCL-loaded DCs treated with DsII extracts at 100 *μ*g/mL were used as stimulator cells. Splenic (a) CD4^+^ and (b) CD8^+^ T cells were obtained from syngeneic mice as responder cells. Ratios of stimulator cells to responder cells were set between 1 : 1 and 1 : 1000. T-cell proliferation activity is represented as fold over control (i.e., T cell only). Data represent the mean ± SE obtained from three independent experiments.

To evaluate the various DsII phytoextracts as vaccine adjuvants, the TCL-loaded DCs were treated with DsII samples prepared from different *Dioscorea *species/strains, and the activity of resultant DCs on T-cell proliferation was tested. DsII-TN5 induced highest proliferation of both CD4^+^ T cells (Figure 1(a)) and CD8^+^ T cells (Figure 1(b)) by TCL-loaded DCs. The splenic T-cell proliferative responses were dose dependent when tested between 1 : 1 and 1 : 1000 (stimulator : responder ratio). Treatments of DCs withDsII-*bat* and DsII-TN1 extracts also significantly enhanced T-cell proliferation, when compared to the control (iDC + TCL).

### 3.2. Determination of Optimal Dose of DsII-TN5 for TCL-Loaded DC Stimulation of Activation of T-Cell Proliferation

**Figure 2:**
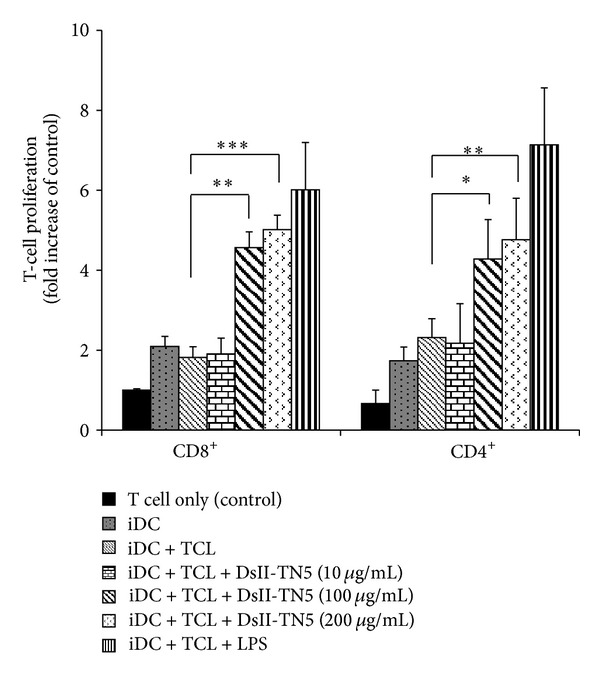
Effect of DsII-TN5 used as an adjuvant with TCL-loaded DC vaccine. TCL-loaded DCs were treated with DsII-TN5 at 10, 100, and 200 *μ*g/mL or with LPS at 1 *μ*g/mL (positive control). Cells were then cocultured with splenic T cells in the ratio 1 : 100 (DCs : T cells). Data represent the mean ± SE obtained from three independent experiments.

TCL-loaded DCs treated with DsII-TN5 (DCs/TCL/DsII-TN5) of varying concentrations dose dependently increased CD8^+^ and CD4^+^ T-cell proliferation (Figure 2). DsII-TN5 at a concentration of 100 *μ*g/mL starts to exhibit a significant stimulatory effect on CD8^+^ and CD4^+^ T-cell proliferation, in comparison with the control group (iDC + TCL). Among all the treatments, iDC + TCL + DsII-TN5 (200 *μ*g/mL) treatment showed the highest level of induction of CD4^+^ and CD8^+^ T-cell proliferation.

We also tested whether high doses of DsII-TN5 affected tested DCs viability. DsII-TN5 at between 0.5 and 5 *μ*g/mL did not impair the viability of tested DCs; interestingly, however, when test concentrations were raised to between 25 and 250 *μ*g/mL, DsII-TN5 significantly increased DC viability (data not shown).

### 3.3. DsII-TN5-Treated Mature DCs Exhibit High Levels of CD40, CD80, and CD86

**Figure 3:**
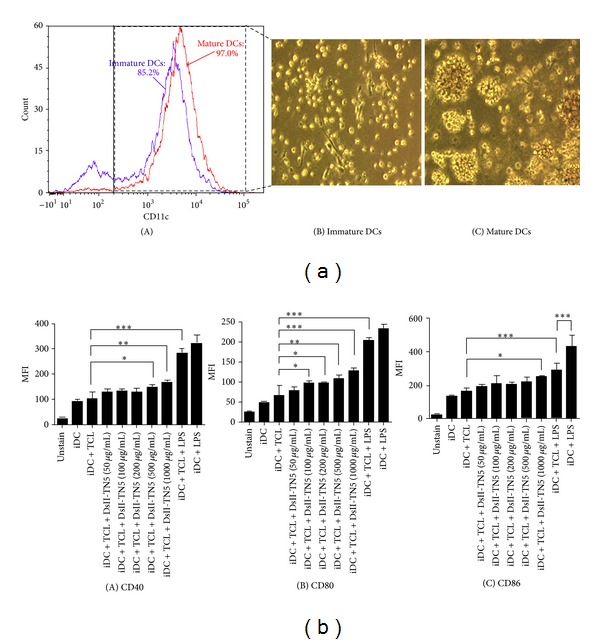
Phenotypic changes in DsII-TN5-treated DCs. (a) The quality of BMDCs was measured by expression of CD11c. On day 7 of cell culture, DCs were cultured in media supplemented with or without 1 *μ*g/mL LPS for 24 h. The CD11c^+^ cell population of DCs was monitored by flow cytometry and DC morphology observed by microscopy. (A) The CD11c^+^ cell population was detected to be 85.2% (blue line) in immature DCs (without LPS treatment) and was detected to be 97% (red line) in mature DCs (with LPS treatment). Morphological characteristics of (B) immature DCs and (C) mature DCs at 400x magnification. (b) Phenotypic changes in (A) CD40, (B) CD80, and (C) CD86 cell-surface marker expressions on TCL-loaded DCs in response to treatment with DsII-TN5 at 50–1000 *μ*g/mL doses. The *y*-axis shows the mean fluorescence intensity (MFI). Data represent the mean ± SD obtained from three independent experiments.

To investigate whether DsII-TN5 can affect the maturation status of DCs, we assayed the phenotypic change of three specific surface markers (i.e., CD40, CD80, and CD86) of CD11c^+^ DCs. LPS, as a TLR4 ligand, served as a positive control for DC maturation. DCs cultured with LPS were used to study the morphological changes between immature and mature DCs. The high quality of the CD11c^+^ DCs as tested cells is shown in Figure 3(a). The population of the CD11c^+^ cells in the immature DC (untreated DC) group was detected to be 85.2% (blue line), and this was substantially increased in mature DCs to 97% (red line) (Figure 3(a)(A)) after tested DCs were cultured in the presence of LPS. We also observed morphological changes in the DCs after LPS treatment. The morphological features of immature and mature DCs are shown in Figures 3(a)(B) and 3(a)(C), respectively. LPS-stimulated DCs are more clustered or aggregated and exhibit longer dendrite extensions than unstimulated DCs. The high quality of CD11c^+^ DCs is shown in Figure 3(a). Expression of CD11c^+^ molecules on immature DCs was detected to be 85.2%; it was substantially increased to 97% in mature DCs (Figure 3(a)(A)). The morphologies of immature and mature DCs are shown in Figures 3(a)(B) and 3(a)(C), respectively.  Figure 3(b) shows that TCL alone had little effect on expression of CD40, CD80, and CD86. However, in combination with DsII-TN5, TCL-loaded DCs exhibited marginally increased expression of CD40 and CD86, and more significantly increased CD80 expression (Figure 3(b)). At 100 *μ*g/mL, DsII-TN5 treatment enhanced the expression level of CD80 to *≈*70%, as calculated by {[(iDC + TCL + DsII-TN5 (100 *μ*g/mL)) − (iDC + TCL)]/[(iDC + TCL) − (unstain)]} × 100% (Figure 3(b)(B)), whereas the expression of CD40 and CD86 markers was only upregulated at 500 and 1000 *μ*g/mL (Figures 3(b)(A) and 3(b)(C)), as compared with the control group (iDC + TCL). In addition, maturation level of tested cells as detected by the three test markers in the iDC + LPS group was apparently higher than that of DCs in the iDC + TCL + LPS group (Figures 3(b)(A), 3(b)(B), and 3(b)(C)). This suggests that TCL treatment can suppress the LPS-induced maturation process in tested DCs. This finding may in part explain the relatively limited DC maturation resulting from treatment with TCLs, which may in turn hinder the immune response resulting from the TCL-loaded DC cancer vaccines.

### 3.4. DsII-TN5 Increases Expression of IL-1*β* and Decreases Secretion of TGF-*β* from Tested DCs

**Figure 4:**
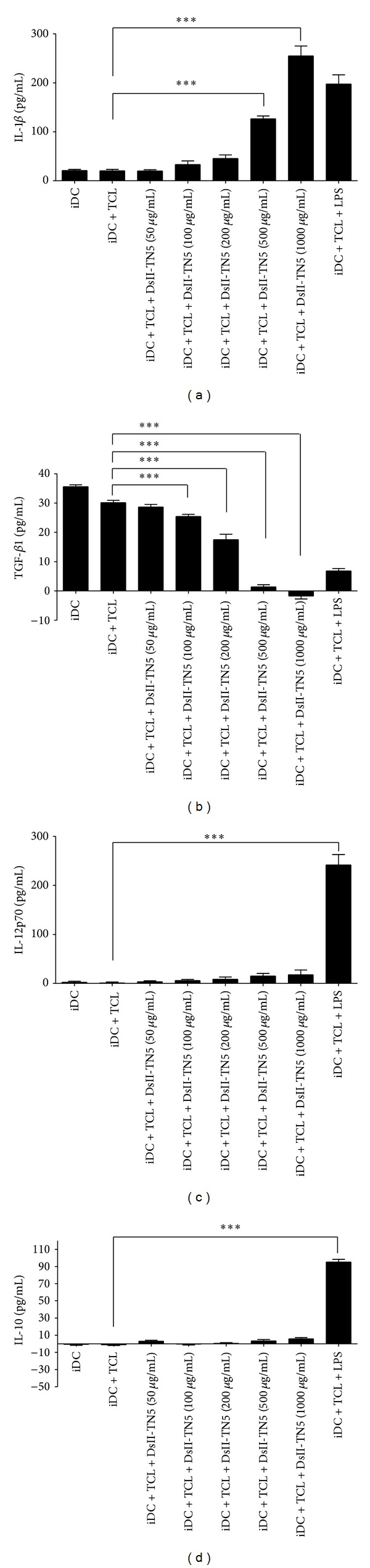
Effect of DsII-TN5 treatment on DC cytokine expression profiles. Expressions of (a) IL-1*β*, (b) TGF-*β*1, (c) IL-12p70, and (d) IL-10 in DsII-TN5- or LPS-treated DCs were analyzed for cytokine secretion by ELISA. Data represent the mean ± SD obtained from three independent experiments.

To further investigate the effect of DsII-TN5 on activation of TCL-loaded DCs, we analyzed the expression profile of specific cytokines in tested DCs. Expression levels of IL-1*β* were increased by DCs/TCL/DsII-TN5 in a dose-dependent manner (Figure 4(a)). In comparison, only a slight, if any, increase in IL12p70 expression was detected in tested cells (Figure 4(c)). In contrast, a substantial decrease in TGF-*β* expression was detected in response to DsII-TN5 (Figure 4(b)). In comparison, no effect on IL-10 expression was detected in DCs treated with DsII-TN5 (Figure 4(d)).

### 3.5. A Specific Regime for TCL Loading and LPS Stimulation of DCs Can Efficiently Enhance Cancer Vaccine Efficiency

**Figure 5:**
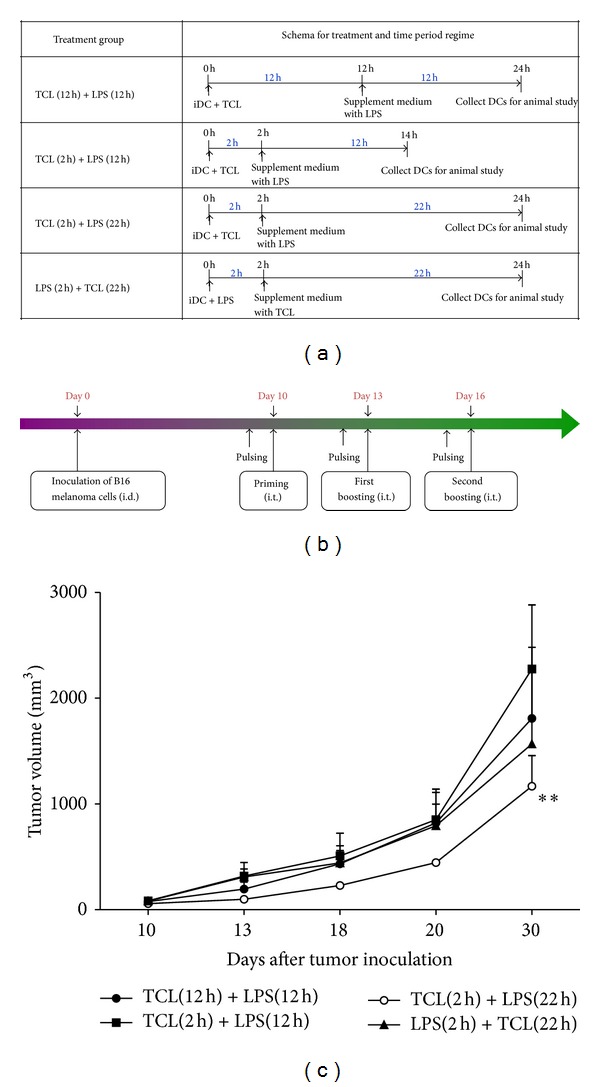
Time course study on incubation of DCs with tumor cell lysate (TCL) accompanied by subsequent stimulation with LPS as adjuvant. (a) Treatments and culture conditions used in the time course study. (b) Schema of experimental design. (c) Comparison of regimes of LPS accompanied by TCL treatment for optimal processing and preparation of DCs vaccines. All data are expressed as mean ± SE.

In an attempt to enhance the antitumor effect of a TCL-loaded DC-based cancer vaccine, we investigated the optimal culture conditions for loading TCL onto DCs and activating DCs using LPS as adjuvant. Four different regimes were used to test stimulation of DCs (Figure 5(a)). The schema for vaccination against B16 melanoma is shown in Figure 5(b). Preparation of DC vaccines using TCL (2 h) + LPS (22 h) treatment was most effective in inhibiting growth of test B16 melanoma (Figure 5(c)); therefore, this regime was chosen for subsequent antitumor immunity studies. 

### 3.6. DsII-TN5 as Adjuvant Effectively Enhances Antitumor Immunity and Suppresses MDSC Activity in a TCL-Loaded DC Vaccine in a Therapeutic Model

**Figure 6:**
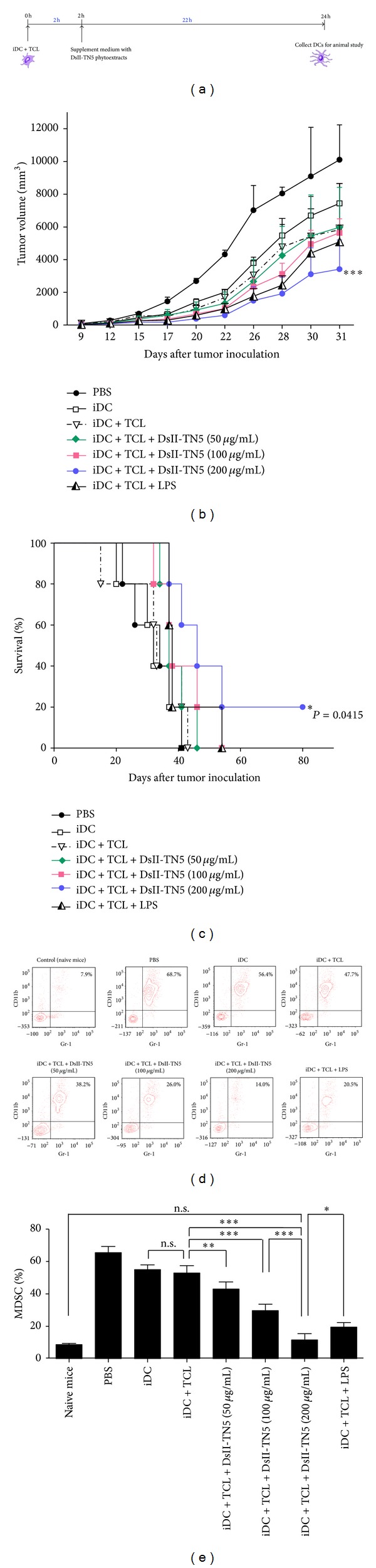
Adjuvant effect of DsII-TN5 on antimelanoma immunity in a mouse model. (a) Schema of preparation of DC-based cancer vaccines. (b) Tumor growth and (c) survival rates in tumor-bearing mice after tumor challenge. All data are expressed as mean ± SE. (d) Percentage of MDSCs (CD11b^+^Gr-1^+^) was analyzed using flow cytometry, calculated, and presented in the bar chart. (e) Data represent the mean ± SD of three replicates.

The above studies suggest that, for possible clinical application, DCs/TCL/DsII-TN5 may activate DC maturation and induce a potent T-cell immune response. We, therefore, next investigated the possible application of TCL-loaded and DsII-TN5-treated DC-based cancer vaccines against primary B16 melanoma (experimental design schema, Figure 5(b); vaccine sample preparation, Figure 6(a)). Some antitumor activity was detected in mice receiving TCL-loaded DCs with no adjuvant treatment as compared to iDC treatment only (Figure 6(b)). The average tumor size on day 31 was reduced (approximately 1600 mm^3^) in test mice vaccinated with iDC + TCL treatment (*≈*5830 mm^3^), as compared with iDC only (*≈*7435 mm^3^). When DsII-TN5 was employed as an adjuvant to the DC-based vaccine, the therapeutic efficacy of the TCL-loaded DC vaccine was significantly increased in a dose-dependent manner. Among the three tested concentrations, the iDC + TCL + DsII-TN5 (200 *μ*g/mL) vaccine group conferred the strongest inhibitory effect on tumor growth and also significantly prolonged survival time in tested mice (*P* = .0415), as compared to the iDC + TCL mice or any of the other groups including the iDC + TCL + LPS group (Figures 6(b) and 6(c)). MDSCs are a major component of the immunosuppressive network present in the tumor microenvironment. MDSCs are also known to markedly expand in tumor-bearing mice [27]. The specific immune cell phenotypic and population changes seen in response to DsII-TN5 adjuvant treatment are shown in Figures 6(d) and 6(e). The MDSC level in test mice in the iDC + TCL + DsII-TN5 (200 *μ*g/mL) group was dramatically decreased to 14%, as compared to 47.7% in the iDC + TCL group. This effect is, therefore, clearly dependent on the dose of DsII-TN5 applied to the DCs, and the optimal effect (14%) at 200 *μ*g/mL DsII-TN5 was even stronger than that (20.5%) detected for LPS (positive control) treatment (Figures 6(d) and 6(e)). Of note, levels of MDSCs in the iDC + TCL + DsII-TN5 (200 *μ*g/mL) group and the naive mice group were quite similar, and the difference was not statistically significant, showing that the 200 *μ*g/mL treatment dosage was highly efficient in suppressing test MDSC activity. In contrast, there was a 2.5- to 8.5-fold difference between the naive and other test treatment groups indicating a considerably reduced effect on suppression of MDSCs. We consider these results to be highly significant, due to the important roles of MDSCs in tumor growth and progression. As a followup, we also observed that the suppressive effect of test DC vaccine regimes on the MDSC population was highly correlatable with the trend observed for the effect on tumor progression (Figures 6(b) and 6(d)). Together, these data demonstrate that DsII-TN5 treatment can inhibit MDSCs in vaccinated mice, and this activity may help mediate the therapeutic immunity achieved by TCL-loaded DC vaccines against melanoma. 

**Figure 7:**
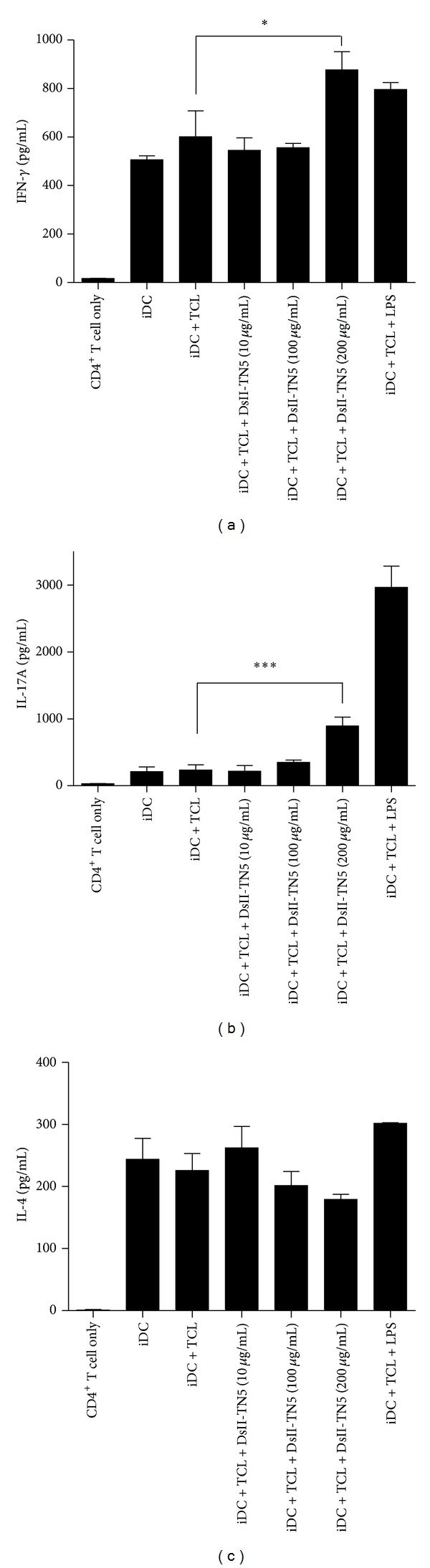
Effect of DsII-TN5-treated DCs on CD4^+^ T-cell polarization. Expressions of IFN-*γ* (a), IL-17A (b), and IL-4 (c) in conditioned media after coculture of DCs and CD4^+^ T cells were analyzed by ELISA. Data represent the mean ± SD obtained from three independent experiments.

In order to examine the possible mode of function(s) of DsII-TN5 on the observed DC-mediated antimelanoma immunity, we investigated the possible polarization effect of DsII-TN5-treated DCs on CD4^+^ T cells. After DsII-TN5 extract treatment, the DsII-TN5-treated DCs were cocultured with CD4^+^ T cells, and the levels of Th1-, Th2-, and Th17-related cytokines in cocultured media were measured by ELISA. The data showed that IL-17A (Th17-type cytokine) and IFN-*γ* (Th1-type cytokine) were significantly augmented in the cocultured medium of the DsII-TN5-treated DC group (iDC + TCL + DsII-TN5 200 *μ*g/mL), as compared with control group (iDC + TCL); however, IL-4 (Th2-type cytokine) level was not or only slightly (*≈*21%) affected in test cells of iDC + TCL + DsII-TN5 200 *μ*g/mL group (Figure 7). These results lead us to suggest that DsII-TN5 extract can activate DCs to enhance subsequent Th1 and Th17 responses, which may further elicit the observed vaccine effect on antitumor activity.

**Figure 8:**
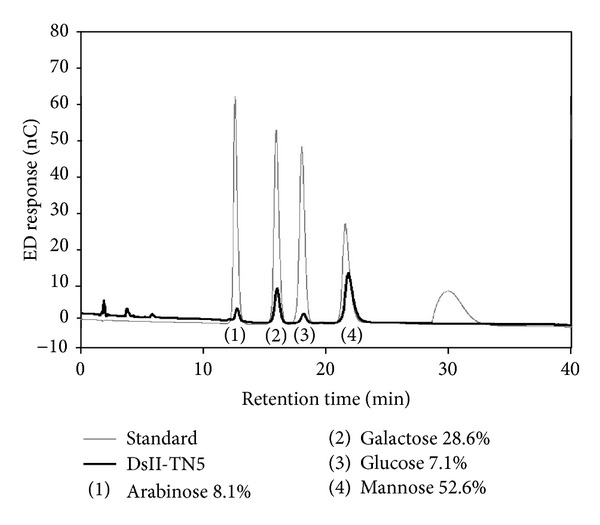
Sugar composition of DsII-TN5 hydrolysate fractions. Separation and quantification of monosaccharide composition of DsII-TN5 (black) and standards (gray) were analyzed by HPAEC. DsII-TN5 is a mixture of polysaccharides containing different sugar residues. Identified monosaccharide composition of DsII-TN5: (1) arabinose (8.1%), (2) galactose (28.6%), (3) glucose (7.1%), and (4) mannose (52.6%). Data represent the mean ± SD of three replicates.

Based on the monosaccharide composition analysis of DsII-TN5, the major moiety of sugar residue compositions of DsII-TN5 were found to be enriched in mannose (52.6%) and galactose (28.6%), with only a minor contribution from arabinose (8.1%) and glucose (7.1%) (Figure 8). This result, along with our findings on DsII [22], suggests that plant polysaccharide species with specific sugar compositions, such as the mannan and galactan of DsII-TN5, may warrant systematic evaluation for use in dendritic cell- (DC-) based cancer vaccines in future biochemical studies.

## 4. Discussion

This study explored the safety, efficacy, and clinical feasibility of a DC-based cancer vaccine adjuvant. We designed a therapeutic cancer vaccine protocol that combined the DC-activating effect of biochemically defined phytoextract from the specific medicinal plant* Dioscorea* with augmentation of specific immunogenicity by TAAs derived from a host tumor. The DsII-TN5 phytoextract, which contains mainly mannose and galactose-enriched polysaccharides, effectively activated DCs by upregulating the expression of specific costimulatory molecules and cytokines. When DsII-TN5 phytoextract was used in TCL-loaded DC-based cancer vaccine preparation, it strongly augmented immunity against melanoma in tested mice. We speculate that these bioactivities may provide a microenvironment that favors the activation of effector T cells, leading to increased efficacy of tested TCL-loaded DC vaccines against tumor cells. An increase in the concentration of DsII-TN5 as an *ex vivo* supplement to the TCL-loaded DC-based vaccine may be beneficial to the efficacy of DC-based vaccine therapy.

PAMPs are active components for adjuvants and confer proinflammatory properties [28]. For high-level recognition of specific PAMPs by PRRs, DCs were apparently stimulated to express high levels of CD40, CD80, and CD86, a process that augments the priming of effector T cells. Previous studies have indicated that bioactive polysaccharides/glycoproteins isolated from *Dioscorea* can activate TLR4 signaling pathways [20, 21]. Treatment with DsII-TN5 induced TCL-loaded DCs to strongly activate T-cell proliferation (Figures 1 and 2) and apparently augmented specific costimulatory expression (Figure 3); we therefore suggest that a combination of TCL and DsII-TN5 may induce PAMPs signals, effectively promoting DCs to express a spectrum of signals required for potent activation of naive T cells. This resulted in the efficacious augmentation of the antitumor effect of the DC-based tumor vaccine. 

DCs can mediate T-cell responses through expression and/or release of specific cytokines, and this mechanism is considered to be a major contributor to the polarization of T cells. Previous studies have reported that both IL-10 and TGF-*β*1 can suppress the effector CD8^+^ T-cell response, promote naive T-cell differentiation into Treg cells, and recruit MDSCs [29, 30], leading to a strong immunosuppressive effect. Furthermore, the tumor microenvironment in general strongly favors the accumulation of Treg cells, the induction or recruitment of MDSCs, and a corresponding increase in expression of TGF-*β*1 and IL-10 [31, 32]. The expression of TGF-*β*1 and IL-10 detected in our study showed that DC/TCL/DsII-TN5 dose dependently inhibited the expression of TGF-*β*1, and the expression of IL-10 was not affected (Figures 4(b) and 4(d). Further, treatment with DC/TCL/DsII-TN5 vaccine suppressed MDSC level in the blood of tested mice (Figures 6(d) and 6(e). We conclude that DsII-TN5 can significantly reduce circulatory MDSCs in the blood of vaccinated tumor-bearing mice, and that this effect could be due to the suppressive effect of DsII-TN5 on secretion of TGF-*β*1 in tested DCs. IL-1*β* is known as a pro-inflammatory cytokine that is crucial for induction of Th17 cells [33, 34], whereas IL-12 favors the differentiation of Th1 cells [35, 36]. Both Th17 and Th1 activities play key roles in tumor rejection [37]. In our study, the DsII-TN5 significantly increased IL-1*β* secretion in tested DCs (Figure 4(a)), but only slightly increased the expression of IL-12p70 (Figure 4(c)). Our data also shows that DsII-TN5 can indeed activate DCs and mediate a subsequent enhancement of Th17 and Th1 responses. IL-17A (Th17-type cytokine) and IFN-*γ* (Th1-type cytokine) were found to be significantly augmented in the cocultured medium of the DsII-TN5-treated DC group (iDC + TCL + DsII-TN5 200 *μ*g/mL), as compared with control group (iDC + TCL); however, IL-4 (Th2-type cytokine) expression was apparently not affected in these tested cells (Figure 7). Taken together, this result suggests that the antimelanoma activity of tested DsII-TN5 vaccine is likely elicited through both Th1 and Th17 responses, but not Th2 cells. 

DCs play an important role in innate and adaptive immune responses. The ability of DCs to prime and expand an immune response is regulated by DC-derived signals. The efficiency of DC-based vaccine against various cancers has been shown to be based mainly on activating T-cell response [38–40]. The activation of T-cell response is decided by specific interaction signals of DCs and T cells. The necessary signals of T-cell activation not only include the expression of DC maturation markers (i.e., CD40, CD80, and CD86) but also the secretion of specific cytokines (i.e., IL-1*β* and IL-12). Although our current results show that DsII-TN5 treatment (200 *μ*g/mL) did not result in a strong stimulation of CD40, CD80 and CD86 expressions in tested DCs, DsII-TN5, effectively increased the IL-1*β* secretion from tested DCs. Subsequently, the DsII-treated DCs apparently promoted Th1- and Th17-related cytokine expression (i.e., IFN-*γ* and IL-17) (Figure 7). These cytokine expression results thus help explain why DsII-TN5 is able to stimulate anticancer activity of the tested DC-based vaccine *in vivo*.

Our previous study showed that the DsII fraction of *Dioscorea* batatas, is a high-molecular-weight polysaccharide that contains a high percentage (*≈*64%) of mannose sugar residues, which enhances murine splenocyte proliferation *ex vivo* and improves regeneration of bone marrow cells *in vivo* [22]. In this study, we also observed that mannose is the major moiety component of DsII-TN5 (*≈*53%) (Figure 8). These findings, thus, provide clues about the mechanisms of action of a spectrum of polysaccharides of DsII phytoextract in mediating key immune cell functions, and these activities may have applications in anticancer vaccines. 

In conclusion, TCLs provide antigenic messages to test DCs and generate antigen-specific responses. Upon DsII-TN5 stimulation, TCL-loaded DCs become fully activated to express costimulatory molecules and secrete specific cytokines, which may result in efficacious retardation of tumor growth and prolonged survival in tested mice. DsII-TN5 may, thus, have potential for further development as an adjuvant for use in TCL-loaded DC vaccines against cancers. This study may also suggest a general strategy for screening and evaluation of phytoextracts as potential adjuvants for DC-based vaccines against malignancies.
